# Delivery of siRNA in vitro and in vivo using PEI-capped porous silicon nanoparticles to silence MRP1 and inhibit proliferation in glioblastoma

**DOI:** 10.1186/s12951-018-0365-y

**Published:** 2018-04-13

**Authors:** Wing Yin Tong, Mohammed Alnakhli, Richa Bhardwaj, Sinoula Apostolou, Sougata Sinha, Cara Fraser, Tim Kuchel, Bryone Kuss, Nicolas H. Voelcker

**Affiliations:** 1 Monash Institute of Pharmaceutical Sciences, Monash University, 381 Royal Parade, Parkville, VIC 3052 Australia; 2 Future Industries Institute, University of South Australia, Mawson Lakes, Adelaide, SA 5095 Australia; 3 Melbourne Centre for Nanofabrication, Victorian Node of the Australian National Fabrication Facility, Clayton, VIC 3168 Australia; 4 School of Medicine, Flinders University, Bedford Park, Adelaide, SA 5042 Australia; 5 South Australian Health and Medical Research Institute, North Terrace, Adelaide, SA 5000 Australia

**Keywords:** Brain tumour, Gene delivery, Nanoparticles, Multidrug resistance-associated protein, siRNA, Cell proliferation

## Abstract

**Background:**

Multidrug resistance-associated protein 1 (MRP1) overexpression plays a major role in chemoresistance in glioblastoma multiforme (GBM) contributing to its notorious deadly nature. Although MRP1-siRNA transfection to GBM in vitro has been shown to sensitise the cells to drug, MRP1 silencing in vivo and the phenotypic influence on the tumour and normal tissues upon MRP1 down-regulation have not been established. Here, porous silicon nanoparticles (pSiNPs) that enable high-capacity loading and delivery of siRNA are applied in vitro and in vivo.

**Result:**

We established pSiNPs with polyethyleneimine (PEI) capping that enables high-capacity loading of siRNA (92 µg of siRNA/mg PEI-pSiNPs), and optimised release profile (70% released between 24 and 48 h). These pSiNPs are biocompatible, and demonstrate cellular uptake and effective knockdown of MRP1 expression in GBM by 30%. Also, siRNA delivery was found to significantly reduce GBM proliferation as an associated effect. This effect is likely mediated by the attenuation of MRP1 transmembrane transport, followed by cell cycle arrest. MRP1 silencing in GBM tumour using MRP1-siRNA loaded pSiNPs was demonstrated in mice (82% reduction at the protein level 48 h post-injection), and it also produced antiproliferative effect in GBM by reducing the population of proliferative cells. These results indicate that in vitro observations are translatable in vivo. No histopathological signs of acute damage were observed in other MRP1-expressing organs despite collateral downregulations.

**Conclusions:**

This study proposes the potential of efficient MRP1-siRNA delivery by using PEI-capped pSiNPs in achieving a dual therapeutic role of directly attenuating the growth of GBM while sensitising residual tumour cells to the effects of chemotherapy post-resection.

**Electronic supplementary material:**

The online version of this article (10.1186/s12951-018-0365-y) contains supplementary material, which is available to authorized users.

## Background

Glioblastoma multiforme (GBM) is a deadly form of brain cancer with only a 5% survival rate at 5 years [1] and the age-standardised mortality rate of brain cancer in 2012 remains the same as in 1982 [2]. The mainstay of therapy is surgical resection. Factors that contribute to the deadly nature of this cancer include the invasiveness of GBM cells, and therefore residual disease, at the resection margins; the selective permeability of the blood–brain barrier (BBB), and the inherent chemoresistance in the endothelial layer at the BBB and in the GBM cells [3, 4]. As the drug fails to penetrate and accumulate, it leads to poor chemotherapy effectiveness in both consolidation and treatment of unresectable tumours.

Chemoresistance results from the expression of membrane-bound efflux transporters, such as the multidrug resistance protein (MRP) superfamily [5]. Multidrug resistance-associated protein 1 (MRP1), a MRP subtype, is a 190 kDa protein, through the hydrolysis of ATP, it actively removes substrates from cytoplasm [6]. It’s overexpression in certain tumours removes drugs from cancer cells compromising treatment effectiveness [7]. Conventional drugs for GBM treatment, such as temozolomide (TMZ) and vincristine (VCR), are substrates of MRP1 which is overexpressed in brain tumours [8] and on the apical surface of endothelial cells of the BBB [9]. These drugs are transported out of the tumour and out of the intracranial space, contributing significantly to the multidrug resistant phenotype of GBM.

Inhibition of MRP1 is a strategy for chemosensitisation and this approach has been substantiated in lung carcinoma in vitro and in vivo [10]. Small molecules are discovered to target and attenuate MRP1 function in various carcinomas over the last decade [11–13]. In comparison, small interfering RNA (siRNA) are more economical, versatile and effective in specific knockdown of protein [14], however its susceptibility to degradation and incapability in penetrating cell plasma membrane are the main obstacles for translation into clinical practice [15]. Nanoparticle delivery is a way to overcome those pharmacokinetic limitations, in which we demonstrated the use of bare porous silicon nanoparticles (pSiNPs) to deliver siRNA into cells [16]. In particular, pSiNPs were used as the delivery vehicle due to their high biocompatibility and degradability, and their degradation product, silicic acid, is non-toxic and is cleared rapidly [17, 18]. The high porosity and surface area of pSiNPs enables high concentrations of therapeutics to be delivered per weight of pSiNP [19, 20]. These pSiNPs have been employed in drug delivery applications such as delivery of enzymes [21], small molecules [22], and nucleotides [23]. The release of the drug can be easily tailored by controlling the degradation rate of pSiNPs and their surface chemistry [24, 25]. Thermal hydrocarbonisation (THC) treatment is a well-established modification to improve the hydrolytic stability of pSiNPs [26–28]. Owing to the polyanionic nature of siRNA, cationic surface treatments are believed to be more favourable to retain siRNA inside pSiNP [29].

MRP1 knockdown in GBM cells in vitro using various polymeric vectors as transfection method has suggested that such anti-chemoresistance approach is viable, however, the inhibition of MRP1 in GBM tumours has not yet been established. Moreover, the side-effect of MRP1 knockdown on phenotypes of GBM cells and on other MRP1-expressing cells has not yet been investigated. Here, we studied the extend of MRP1 silencing and associated effects using siRNA delivered in pSiNPs with PEI optimised capping to control its release, and also report the effect and mechanism of this on the inhibition of proliferation of GBM cells in vitro and in vivo. At the same time, we also evaluated the side effects of MRP1 silencing in the organs that the nanoparticles may accumulate in. We anticipate that the results will provide insights into the potential of MRP1 gene therapy in GBM treatment, and unlock the door to eradication of this fatal disease.

## Methods

### Porous silicon nanoparticle (pSiNP) fabrication

pSiNPs were fabricated according to the previously reported procedure [23, 27] from p+ type (0.01–0.02 Ω cm) silicon wafers (Siegert Consulting Co., Aachen, Germany) by periodically etching at 50 (2.2 s period) and 200 (0.35 s period) mA/cm^2^ in a solution of 1:1 hydrofluoric acid (38%):ethanol (EtOH) for 20 min. Afterwards, the porous silicon (pSi) films were detached from the substrate by abruptly increasing the current density to electropolishing conditions (250 mA/cm^2^, 3 s period). The detached multilayer pSi films were then thermally hydrocarbonised under N_2_/acetylene (1:1, vol.) flow at 500 °C for 15 min, and cooled down to room temperature under a stream N_2_ gas. Subsequently, pSiNPs were produced by wet ball-milling (ZrO_2_ grinding jar, Pulverisette 7, Fritsch GmbH, Idar-Oberstein, Germany) of the thermally hydrocarbonised pSi films in 1-decene. The completion of THC surface treatment was confirmed by FTIR analysis via the disappearance of Si-H_x_ species (2200 cm^−1^) and strong signal corresponding to C–H (3000 cm^−1^) and Si–C (770 cm^−1^) species (Additional file 1: Figure S1). pSiNPs were harvested by centrifugation (1500×*g*, 5 min). pSiNP stock solutions were prepared in EtOH at 20 mg/ml.

### siRNA loading, PEI capping and release kinetics

After pSiNP fabrication and characterisation, siRNA was loaded and particles were coated with polyetherimine (PEI). Both test siRNA (siRNA) and scramble siRNA (ctrl siRNA) were purchased from GenePharma Co. Ltd. (Shanghai, China). siRNA is complementary to mRNA sequence encoding for MRP1 (5′ GAGGCUUUGAUCGUCAAGUTT 3′), while ctrl siRNA sequence (5′ UUCUCCGAACGUGUCACGUTT 3′), lacks homology to all other genes. These siRNAs were solubilised in molecular biology grade water to make stock solutions (500 µg/ml). siRNA loading was performed by our previously established protocol [23]. Briefly, siRNA (100 µl of 500 µg/ml in water) was added to pSiNPs (250 µl of 1 mg/ml in EtOH), mixed by sonication for 1 min, then incubated overnight at 4 °C. Following the incubation, the loaded pSiNPs were isolated by centrifugation at 5000 rcf at 4 °C then resuspended in EtOH and centrifuged two more times to wash off unbound siRNA. To cap the siRNA loaded pSiNPs with PEI, the pellet was then resuspended in 0.05% PEI of 25 kDa (Sigma-Aldrich, 408727) diluted in EtOH and incubated for 20 min. pSiNPs were centrifuged, and the pellet was washed with EtOH for three times to wash off excess PEI.

To measure the loading efficiency of siRNA into pSiNPs, siRNA concentration in the supernatant was measured at 260 nm by using a Nanodrop spectrophotometer (ND2000, ThermoFisher) and the amount of siRNA was then back calculated. The loading efficiency (%) = (the siRNA amount in loading buffer − the siRNA amount in supernatant)/(the siRNA amount in loading buffer) × 100%.

The release kinetics were determined by incubating PEI-pSiNPs/siRNA (MRP1 and ctrl) in PBS at 37 °C for 120 h. The supernatant was extracted at designated time points, and the amount released was back calculated from the absorbance measurement at 260 nm. The in vitro and in vivo treatment schedule was based on the release kinetics. The reason for conducting release kinetic assay in PBS, instead of culture medium is that quantitating siRNA released in protein containing medium is problematic owing to the masking of the RNA absorbance at 260 nm. Quantitation in PBS is reliable and its physiological relevant pH and osmolality are suitable for demonstrating the siRNA release from PEI-capped pSiNP.

To compare the siRNA knockdown efficacy and to delineate the cell phenotypes influenced by the nanoparticle of choice, lipofectamine (Invitrogen, 11668-019) was used in parallel as a delivery agent. The loading of siRNA to form a siRNA-lipid complex with lipofectamine was performed following the supplier’s protocol. Briefly, siRNA was first diluted in Opti-MEM (Gibco, 51985091). It was then mixed with lipofectamine at a 1:1 ratio to form siRNA-lipid complex. All siRNA loadings were carried out immediately prior to cell exposure under sterile conditions.

### Size and zeta-potential characterisation of pSiNPs

To reveal the size distribution and zeta-potential of pSiNPs, the particles were first washed and resuspended into MilliQ and sonicated for 5 min. The particles were then analysed using a Zetasizer Nano ZS (Malvern, Worcestershire, UK). The analysis was carried out at a scattering angle of 90° at a temperature of 25 °C.

### TEM characterisation

Transmission electron microscopy (TEM) was done to visually examine the size and structure of pSiNPs prepared. pSiNP stock solution was first sonicated to homogenise particles. PEI-pSiNPs and pSiNPs without coating, siRNA-loaded or empty were diluted in EtOH. 10 µl of solution was spotted onto each 300 mesh Cu grid (ProSciTech Co.) and air-dried. The samples were then observed under a field-emission TEM (JEOL, JEM-2100F, Japan). The average pore size of pSiNP was determined firstly by SEM imaging freshly etched pSi film before detachment and ball milling. TEM images was taken to further confirm the pore size by measuring pores of pSiNP. The pore diameter is determined by measuring wall-to-wall distance of individual pores using ImageJ. The average was taken from 50 measurements of randomly selected particles.

### Cell culture

In this study we used U87 cells of human origin that stably express cytoplasmic mCherry, a kind gift from Bakhos A. Tannous, MGH, Massachusetts, USA, and T98G cells of human origin (ATCC^®^ CRL-1690™). Both are well-recognised model cell lines representing GBM, mCherry expressing GBM cells allow live cell imaging of nanoparticle uptake. It is documented that both cell lines demonstrate a similar cell growth rate [30]. Cell lines were maintained in Dulbecco’s Modified Eagle Medium (DMEM, Sigma-Aldrich D6546) supplemented with 10% foetal bovine serum (Invitrogen), 1% antibiotic–antimycotic (Invitrogen) at 37 °C and 5% CO_2_ in a humidified incubator. For lipofectamine-based siRNA delivery, Opti-MEM reduced serum medium was used in culture. For tumour xenograft inoculation preparation, 10 mM HEPES buffer (Sigma-Aldrich, H0887) was supplemented in DMEM for stabilising the pH of high confluence culture. All experiments were conducted following at least two passages after thawing cells.

For the functional inhibition assay, MK-571, the MRP1 inhibitor (a leukotriene receptor ligand), was used to determine the functional activity of MRP1 in T98G GBM cell line, followed by a Calcein-AM (Santa Cruz Biotechnology, Inc., Dallas, TX, USA) accumulation assay [31]. Calcein AM was used to measure the multidrug transporter-mediated activity. It is a non-fluorescent probe, but when it permeates the cells, it hydrolyses to a fluorescent molecule. Cells were seeded at 3 × 10^5^ cells/well in 6 well-plates and allowed to adhere overnight. Subsequently, a total of 25 µM MK-571 was added to each well and incubated for a further 72 h. The cells were analysed at 24, 48, 72 h. At the 24 h time point, cells were incubated with 0.20 µM of Calcein AM staining solution at 37 °C. After incubation for 30 min, cell samples were immediately washed and analysed by Accuri-C6 flow cytometer (BD Accuri Cytometers Inc., USA) to determine cellular uptake of Calcein AM. The control experiment was performed under identical conditions. 1 × 10^4^ events were collected by flow cytometry. Cell viability was measured by trypan blue and Annexin V as described previously [32].

The cells were seeded into 6-well plates at a density of 5 × 10^4^ cells/cm^2^ and maintained in DMEM for 24 h after which the cultured cells were exposed to sterilised pSiNPs (bare/capped with PEI/loaded with siRNA) at a concentration of 0.125 mg/ml (250 µl per well of 1 mg/ml) for 24 h. After 24 h, cells were washed with sterile PBS and a second dose of pSiNPs was added (0.125 mg/ml) for another 24 h, followed by cells washed with sterile PBS then maintained in DMEM for another 24 h before harvesting for different studies.

### Cellular uptake of pSiNPs

To visualise the uptake of pSiNPs in U87 cells, nanoparticles fabricated as described above, were fluorescently labelled with fluorescein-5-thiosemicarbazide. pSiNPs were first hydrosilylated to obtain an activated carboxyl group on the surface by incubating with neat undecylenic acid (UA) for 16 h at 120 °C [33]. The pSiNPs were extracted from UA via centrifugation after the reaction and washed with EtOH five times to ensure complete removal of excess UA. Next, the carboxy-functional pSiNP surface was covalently modified with fluorescein-5-thiosemicarbazide dye by means of ethyl(dimethylaminopropyl)carbodiimide (EDC)/*N*-hydroxysuccinimide (NHS) coupling reaction. EtOH was removed from UA-functionalised pSiNPs by centrifugation and the particles were rinsed with DMF twice. An 8 mM solution of EDC in anhydrous DMF was prepared and 0.2 equivalents of triethylamine were added to the solution. In the meantime, 8 mM solution of NHS was prepared in anhydrous DMF. 500 µl of each solution was removed, mixed well and added to the UA-functionalised pSiNPs, followed by addition of 1 ml of fluorescein-5-thiosemicarbazide solution (4 mM). The entire reaction mixture was allowed to react for 24 h at RT in the dark with stirring. Excess cross linking agent, dye and DMF were removed by centrifugation and the nanoparticles were copiously washed with cold water, followed by an EtOH wash and stored in EtOH. An alternative of tracking the subcellular delivery of siRNA is by loading pSiNP with FAM-labelled siRNA. However, this is not reported here since the change of physical/chemical properties of siRNA and the resulting changes in release profile is unknown.

After fluorescence labelling, pSiNPs were either coated with PEI as described above, or left uncoated. The zeta-potential, hydrodynamic size and release profile of fluorescence labelled pSiNPs/PEI capped pSiNP were unaltered. U87 cells were then exposed to these particles for 3 h, then washed three times with warm PBS to remove excess pSiNPs. Cells were cultured for another 24 h before fixation, counterstained, mounted and imaged under a confocal microscope (ELYRA superresolution microscope, Zeiss Elyra PS.1).

### Trypan Blue viability assay

A Trypan Blue exclusion assay was performed to study the effect of PEI-pSiNP delivery on the viability of cells. Briefly, U87 cells exposed to PEI capped or bare pSiNPs were washed, trypsinised, diluted, and mixed 1:1 with sterile filtered 0.4% Trypan Blue solution (T8154, Sigma). After 5 min incubation, cells were counted using a haemocytometer under a bright field microscope. The proportion of the viable cells to total cells are reported.

### EdU assay of cells

The proliferation rate of U87 cells receiving siRNA delivery by lipofectamine or PEI-pSiNPs, and respective controls were assessed through pulse labelling of cells using the Click-iT EdU Imaging Kit (Invitrogen, C10337). Briefly, cells were seeded on the poly-l-lysine coated coverslips at a density of 5 × 10^4^ cells/cm^2^. At 24 h post exposure, cells were incubated with 10 mM of EdU (5-ethynyl-2-deoxyuridine) in DMEM for 1 h. Cells were then washed and fixed in 4% PFA and permeabilised in 0.5% Triton X-100 in PBS. S-phase cells, which are EdU positive, were visualised following the protocol specified by the manufacturer. All cells were counter-stained by Hoechst 33342, and mounted on a glass slide with fluorescence mounting medium (Dako, S3023). Images were taken under widefield Olympus IX83 fluorescence microscope using a 10× objective. Image analysis was done using Fiji ImageJ v.2.0.0. to automatically count nuclei with positive staining. The averaged ratio of EdU positive nuclei to the total number of cells is reported as “EdU positive nuclei proportion (%)”. This directly reflects the proliferation rate of cells.

### Immunofluorescence

The cells were seeded onto poly-l-lysine coated glass coverslips, in a 6-well plate, at the cell density described, and were exposed to PEI-pSiNPs with or without siRNA, or lipofectamine carrying siRNA, or left untreated. After 24 h, cells were fixed for 10 min with 4% freshly prepared PFA at room temperature, quenched with 100 mM glycine in PBS for 15 min and washed twice with PBS before permeabilisation in 0.05% Triton X-100 in PBS for 5 min at room temperature. After permeabilisation, the cells were washed twice with PBS and blocked with 3% bovine serum albumin (BSA) in PBS for 30 min at room temperature. The cells were then incubated with primary antibodies overnight, washed, and incubated with fluorochrome-conjugated secondary antibodies for 60 min. The slides were washed in PBS then counterstained for 15 min. The primary antibody used was mouse anti-ki67 (Abcam, ab8191). The secondary antibody used was Alexa 488 goat anti-mouse (Invitrogen, A11001). Rhodamine-phalloidin (Invitrogen, R415) and Hoechst (Sigma-Aldrich, B2261) were used to counter label the actin filaments and nuclei, respectively. After washing, cells on coverslips were mounted on a glass slide. These samples were then imaged at high magnification using a confocal microscope (ELYRA Superresolution Microscope, Zeiss Elyra PS.1).

For statistical analysis, images were taken under a widefield Olympus IX83 fluorescence microscope, and counted using the image analysis technique described above. The averaged ratio of ki67 positive nuclei to the total number of nuclei was calculated, directly indicating the progression of the cell cycle.

### Western blotting

To study the relative protein expression of MRP1 and cell cycle checkpoints, protein lysates were analysed by Western blotting. The cells that were exposed to PEI-pSiNPs with MRP1 siRNA or control siRNA, or siRNA delivered in lipofectamine, or left untreated were washed in warm PBS and directly lysed in a sodium dodecyl sulphate (SDS) lysis buffer (2% SDS, 50 mM Tris pH 6.8) supplemented with protease inhibitor cocktail (Sigma, P8340). Tissue samples were thawed and homogenised using Navy Bead Lysis Kits using a tissue homogeniser (Bullet Blender, BB24-AU). The protein concentration was quantified by an EZQ protein quantitation kit (Invitrogen, R3320). The proteins were then mixed with Laemmli loading buffer (BioRad, 161-0747) with β-mercaptoethanol, and boiled at 95 °C for 10 min. To analyse MRP1, proteins were electrophoresed in 10% bis-acrylamide gels, and transferred onto nitrocellulose membranes overnight. For pCdk2 and pHistone H3 analysis, electrophoresis was performed with 14% bis-acrylamide gel. Membranes were blocked with 5% (w/v) non-fat milk powder (Devondale) in PBST (0.05% Tween-20 in PBS) for 60 min, followed by washing with PBST and incubation with a primary antibody in blocking buffer overnight with rocking at 4 °C. The primary antibodies used were mouse anti-MRP1, rabbit anti-Cdk2 pTyr15, and rabbit anti-Histone H3 pSer10 (Abcam, ab24102 and ab136810, respectively). The secondary antibodies used were horseradish peroxidase conjugated Immun-Star goat anti-mouse immunoglobulin G (BioRad, 1705047) and goat anti-rabbit immunoglobulin G (Agrisera, AS09602). The membranes were washed and incubated with the secondary antibodies for 60 min. After washing, membranes were developed with Clarity™ Western ECL Substrate (BioRad, 1705061) and imaged by using a Chemi-Doc with a cool CCD camera (Syngene, G:BOX Chemi XRQ). Expression was measured by densitometry analysis of the bands using Fiji ImageJ, deducting the background intensity, and normalised to the integrated density of the β-actin housekeeping protein band. Knockdown efficiency was calculated as test group/control group x 100%.

To illustrate the distribution of the cell cycle population upon exposure to PEI-pSiNPs with MRP1 siRNA, cells were analysed using flow cytometry, and a histogram of DNA content plotted. Briefly, the exposed cells were trypsinised, PBS rinsed, and fixed dropwise in 100% ethanol. The cells in single cells were rinsed and stained with PI (50 µg/ml), and subsequently analysed using ImageStream flow cytometer (Amnis). Cell populations is gated and measured using Amnis software.

### Mice tumour model

To assess the siRNA delivery, MRP1 knockdown and its subsequent influence on the proliferative state, and the effect of knockdown on distal organs, a subcutaneous xenograft tumour model was established using nude mice. Animal procedures were performed according to a protocol approved by South Australian Health & Medical Research Institute Animal Ethics Committee (Approval number, SAM#98). U87 cells were trypsinised, washed with warm PBS and counted. 5 × 10^6^ cells in chilled PBS were then mixed 1:1 with Matrigel (E1270, Sigma) to a total volume of 100 µl. CD-1 nude mice of mixed gender, between 6 and 8 weeks of age, were then subcutaneously inoculated with the prepared cells on both flanks. Food and water were provided ad libitum, and since CD-1 nudes are immunocompromised, they were housed in individually-ventilated cages (IVC). On week 4 post-inoculation, mice bearing tumours reaching 250 mm^3^ were paired into groups to receive either pSiNPs/Saline, pSiNPs/ctrl siRNA, or pSiNPs/siRNA (MRP1). Each group and time point contained at least two mice, each bearing (four tumours total per treatment). No statistical difference in tumour size was observed between the groups (data not shown). Each group of mice received 2 intravenous doses delivered 24 h apart. Each mouse received 31.25 mg/kg of pSiNPs loaded with siRNA, or the same amount of unloaded pSiNPs, per dose. Mice were humanely killed at 24, 72 h post-treatment. Tumours, kidneys and duodenums were harvested. For histological analysis, tissues were immediately fixed in neutral buffered formalin (NBF, Sigma-Aldrich) at 4 °C for 2 d, followed by paraffin embedding. For protein level analysis, tissues were immediately cut into small cubes, 1 mm^3^, immersed in SDS sample buffer, and snap-frozen. For mRNA level analysis, tissues were immersed into RNAlater (AM7023, ThermoFisher) and stored at − 20 °C.

### Quantitative real time-polymerase chain reaction (qRT-PCR)

MRP1-mRNA silencing was studied using a BioRad CFX Connect QRT-PCR detection system with a SYTO9 reagent to detect the level of MRP1-mRNA expression. In summary, tissue sections were homogenised using TRI Reagent^®^ (Sigma-Aldrich) (1 ml per 50–100 mg of tissue). TissueLyser II Qiagen (QIAGEN^®^, Hilden, Germany) was used to disrupt and homogenise tissue. Total mRNA was extracted using TRI Reagent, according to the manufacturer’s protocol. cDNA was synthesised from mRNA using M-MLV H (−) reverse transcriptase (Promega Corporation, Alexandria, NSW, Australia). The coincident measurement of the “housekeeping” gene glyceraldehyde-3-phosphate dehydrogenase (GAPDH) was used to control the experimental variations of RNA and to normalise the MRP1-mRNA expression data, which was calculated using the ΔΔCt method. The primers were designed via the web tool (http://www.ncbi.nlm.nih.gov). All primers used for QRT-PCR of MRP1-mRNA are shown in Table 1.

**Table 1:** Sequences of the primers used in qRT-PCR analysis

Primer ID		
MRP1_Human	Forward	5′ → AAGGAATGCGCCAAGACTAG → 3′
	Reverse	5′ → CCTTAAACAGAGAGGGGTTC → 3′
GAPDH_Human	Forward	5′ → GTGAAGGTCGGAGTCAACGG → 3′
	Reverse	5′ → TGGAGGGATCTCGCTCCTGG → 3′
MRP1_Mice	Forward	5′ → TGCAGAGGCATCTCAGCAACTC → 3′
	Reverse	5′ → TTCGGCTATGCTGCTGTGTT → 3′
GAPDH_Mice	Forward	5′ → CGACTTCAACAGCAACTCCCACTCTTCC → 3′
	Reverse	5′ → TGGGTGGTCCAGGGTTTCTTACTCCTT → 3′

### Immunohistochemistry

Histological sections were immunohistochemically stained to visualise the MRP1 expression and to study the proliferative state of the tumours. Microtome sectioned tissue specimens of 5 µm were dehydrated at 50 °C overnight, followed by deparaffinisation and rehydration. Rehydrated slides were first incubated in sodium citrate buffer pH 6 at 95 °C for 30 min for antigen retrieval and cooled down to room temperature. For ki67 staining, slides were additionally incubated with 0.1% Triton X-100 in PBS for 10 min to permeablise the nuclear envelope. Slides were then incubated in 1% H_2_O_2_ for 15 min to quench endogenous peroxidase activity, followed by serum blocking for 1 h. Goat anti-mouse IgG Fab (Abcam, ab6668) was added for 1 h to block endogenous IgG. After washing in PBST, slides were incubated with primary against ki67 (Abcam, ab8191) or primary against MRP1 (Santa Cruz, sc-18835) overnight in a moisture chamber at 4 °C. Slides were then incubated with biotinylated mouse-rabbit polyvalent secondary (Abcam, ab64264), followed by streptavidin peroxidase (Abcam, ab64264) incubation. After washing, slides were developed using 3,3′-diaminobenzidine (DAB) substrate for 15 min then counterstained with Mayer’s haematoxylin QS (Vector, H-3404), and eventually mounted in coverslips with DPX.

### Hematoxylin and Eosin (H&E) staining

To study the side-effect of MRP1 knockdown in distal organs, kidney and duodenum were excised and sectioned for histological characterisation. Deparaffinised and rehydrated sections were stained with Lillie-Mayer’s H&E (Australian Biostain P/L) following standard protocols. H&E and immunohistological stained tissue sections were imaged using a bright-field microscope.

### Statistics

Experiments were conducted in triplicate unless otherwise stated. Error bars presented in charts equal ± 1 standard deviation (SD). Statistical differences were tested by non-parametric Kruskal–Wallis 1-way ANOVA test. The hypothesis was accepted at a 95% significant level (p < 0.05).

## Results

### Characteristics of pSiNPs and siRNA release from pSiNPs

**Fig. 1:**
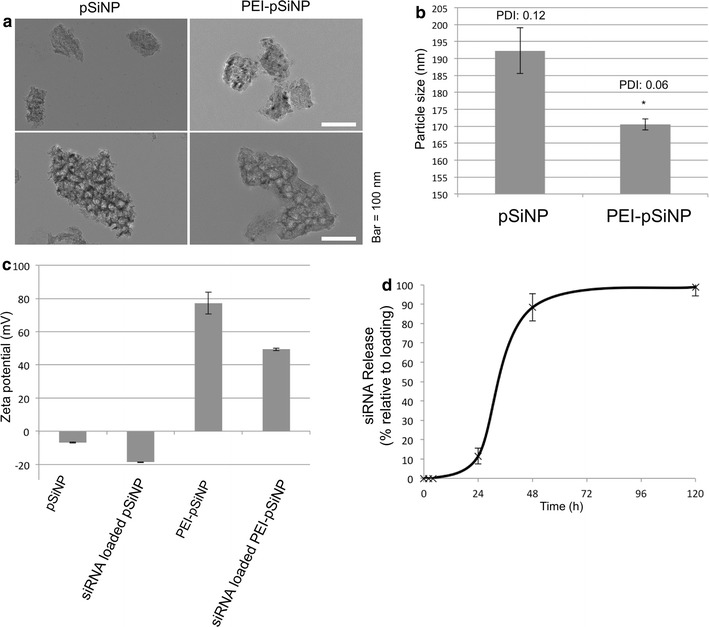
Characterisation of pSiNPs for siRNA delivery. **a** The structure of pSiNPs and PEI-capped pSiNPs under TEM. **b** Average hydrodynamic diameter in MilliQ. **c** Zeta potential of empty pSiNPs and pSiNPs loaded with siRNA. **d** siRNA release kinetics of PEI-coated pSiNPs. (*n *=* 3*; *mean ± standard deviation*; *p < *0.05*)

pSiNPs were fabricated as a vehicle to deliver siRNA for MRP1 silencing. The cellular uptake and biodistribution of the siRNA loaded pSiNP is dependent on the size and surface characteristics [34]. Their size, pore structure and colloidal stability were characterised by means of TEM, dynamic light scattering (DLS), and a zeta-potential analyser. TEM images show that plate-shaped pSiNPs were successfully fabricated at the desired size of approximately 110 nm (Fig. 1a). These particles contained pores of approximately 15 nm in size. The achieved pore dimensions were suitable for loading siRNAs, which are approximately 7.5 nm long and 2.5 nm wide [35, 36]. Judging from TEM images, there was no observable difference in size between PEI-coated and non-coated pSiNPs. The hydrodynamic diameter distribution of these particles was further analysed using DLS in aqueous media (Fig. 1b). This confirmed that particle size distribution was small, where 70% of pSiNP sized between 186 and 198 nm (PDI: 0.12), and 70% of PEI-pSiNP sized between 169 and 173 nm (PDI: 0.06). The PEI-pSiNPs were statistically smaller than the uncoated pSiNPs, and this was attributed to the improved colloidal stability of PEI-pSiNPs in an aqueous medium. To investigate this phenomenon further, we determined the surface zeta potential of pSiNPs. Fabricated pSiNP had a slightly negative zeta potential of − 7 mV (Fig. 1c). As expected, PEI-capped pSiNPs had an overall positive charge, with zeta-potential reaching 80 mV. Loading with siRNA resulted in both types of particles becoming more negatively charged with − 20 and 50 mV in pSiNPs and PEI-pSiNPs, respectively. Nanoparticles with a zeta-potential of more than ± 30 mV are predicted to be colloidal stable [37, 38], which explains the higher stability of PEI-pSiNPs.

Based on the colloidal stability and the advantage in facilitating cellular uptake, we chose PEI-capped pSiNPs for further study. siRNA was loaded into pSiNPs and capped with PEI, and the loading efficiency was calculated as 70 ± 9%, effectively carrying 23 µg of siRNA per 250 µg of PEI-pSiNPs. This loading capacity is much higher than pSiNP loading documented without cationic PEI capping, which was only 1.75 µg per 250 µg of pSiNPs [16]. The release of siRNA in PBS was followed for up to 120 h (Fig. 1d). Substantial siRNA release was observed between 24 and 48 h, where 70% siRNA was released within this period. The release plateaued from 72 h onwards. These kinetics indicated a suitable release profile that allowed uptake and accumulation of PEI-pSiNPs in tumours before releasing most of the siRNA [39].

### Intracellular siRNA delivery by pSiNPs

**Fig. 2:**
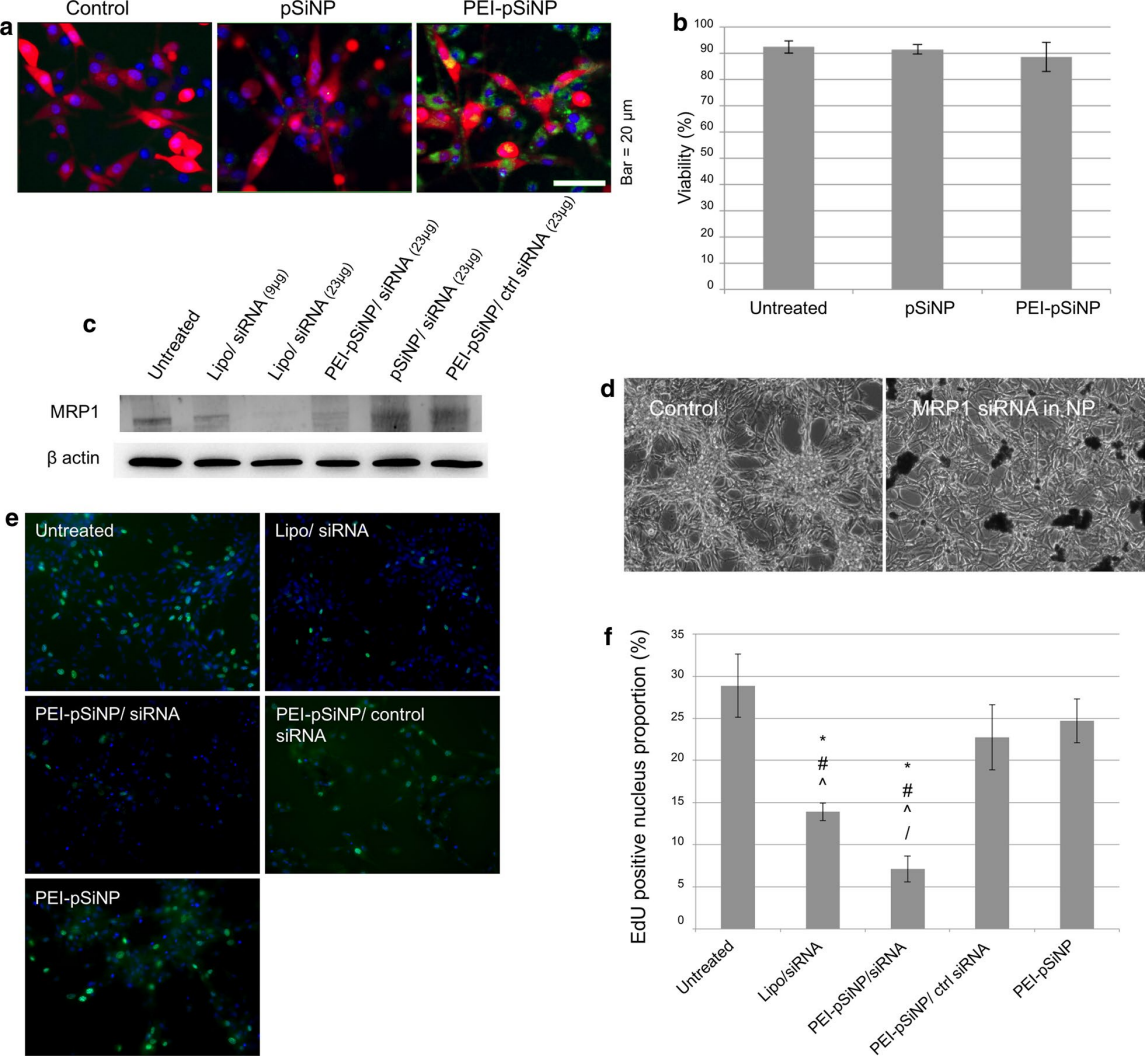
Cellular uptake of pSiNPs and subsequent phenotypic changes in U87 GBM cells. **a** Cellular uptake of fluorescein labelled pSiNPs. Green: pSiNPs; red: cytoplasmic mCherry; blue: nucleus. **b** The viability of U87 cells exposed to pSiNPs measured by Trypan Blue exclusion assay. **c** MRP1 expression in U87 cells exposed to siRNA via lipofectamine (Lipo/siRNA) or nanoparticle (pSiNP) delivery by immunoblotting. **d** Phase contrast image of U87 cells exposed to PEI-pSiNPs carrying MRP1 siRNA and untreated at day 3 post-exposure. **e** The proliferation of U87 cells as measured by EdU labelling of cells in S-phase. Green: EdU positive nuclei; Blue: nuclei. **f** Quantitation of S-phase cell proportions. (*^, #, ^, /^*p *< *0.05 as compared to untreated, PEI*-*pSiNPs/ctrl siRNA*, *PEI*-*pSiNPs and Lipo/siRNA*, *respectively*)

After confirming that the surface chemistry and siRNA release profile of PEI-pSiNPs were suitable, pSiNPs were fluorescently labelled and exposed to the U87 GBM cell line to characterise cellular uptake. As anticipated, greater uptake was observed for positively charged PEI-capped pSiNPs as compared to uncapped pSiNPs (Fig. 2a). Although PEI-capping benefits the loading, favourable release profile, and cellular uptake, it is essential that the nanoparticle delivery by itself is biocompatible to cells. The viability of U87 cells exposed to both types of pSiNPs by Trypan Blue exclusion showed no significant difference between untreated cells and cells exposed to pSiNPs or PEI-pSiNPs (Fig. 2b). This indicate that the cytotoxicity of PEI-capped pSiNPs is minimal and that these particles are fairly biocompatible.

### In vitro MRP1 silencing

Next, we employed PEI-pSiNPs to deliver MRP1 siRNA into U87 cells to study downregulation of MRP1 via immunoblotting. For comparison, we used lipofectamine(Lipo)-based transfection. Results illustrate that the MRP1 expression was downregulated in cells transfected with MRP1 siRNA as compared to untreated controls, with 51 and 30% downregulation in Lipo/siRNA (23 µg siRNA) and PEI-pSiNP/siRNA (23 µg siRNA) treated cells, respectively (Fig. 2c). In contrast, control siRNA delivered via PEI-pSiNPs did not knock down MRP1 in U87 cells, ruling out non-specific effects of the siRNA and of its delivery system. Additionally, the extent of downregulation was dependent on the concentration of siRNA delivered as the cells receiving less Lipo/siRNA (9 µg) expressed more MRP1 as compared to 23 µg delivered via lipofectamine and PEI-pSiNPs (Fig. 2c). To demonstrate the effect of MRP1 downregulation by PEI-pSiNPs/siRNA on drug efflux, we exposed U87 cells to either DOX, or PEI-pSiNPs/siRNA, or co-treatment, or left untreated. Result showed that MRP1 silencing using PEI-pSiNP/siRNA sensitised U87 cells to DOX (Additional file 2: Figure S2). This indicates PEI-pSiNPs delivering MRP1 siRNA is a viable approach to reduce chemoresistance.

Prolonged culture of U87 cells transfected by PEI-pSiNPs/siRNA for 2 more days, resulted in an observable reduction in total cell population as seen in a representative phase contrast image (Fig. 2d). However, such effect of MRP1 on cancer cell growth has not been well documented. We speculated that MRP1 knockdown attenuates cell proliferation in parallel to chemosensitisation. To quantitatively study cell proliferation, U87 cells transfected with siRNA were pulse-labelled by EdU to identify the population of U87 cells that entered S-phase of the cell cycle at a given time (Fig. 2e). Significantly less EdU positive nuclei were observed in Lipo/siRNA and PEI-pSiNP/siRNA treated cells as compared to untreated or PEI-pSiNP/ctrl siRNA treated cells (Fig. 2f). Although there was also a reduction in proliferation rate in PEI-pSiNP and PEI-pSiNP/ctrl siRNA treated cells as compared to untreated cells, this effect was not significant (p = 0.127). In particular, the EdU proportion of cells receiving siRNA delivered by PEI-pSiNPs was significantly lower than for cells receiving siRNA in lipofectamine (p = 0.034). Collectively, these results indicate a decrease in proliferation rate of GBM cells with MRP1 knockdown by siRNA, which this effect was independent of the siRNA delivery method.

### MRP1 functional inhibitor MK-571

**Fig. 3:**
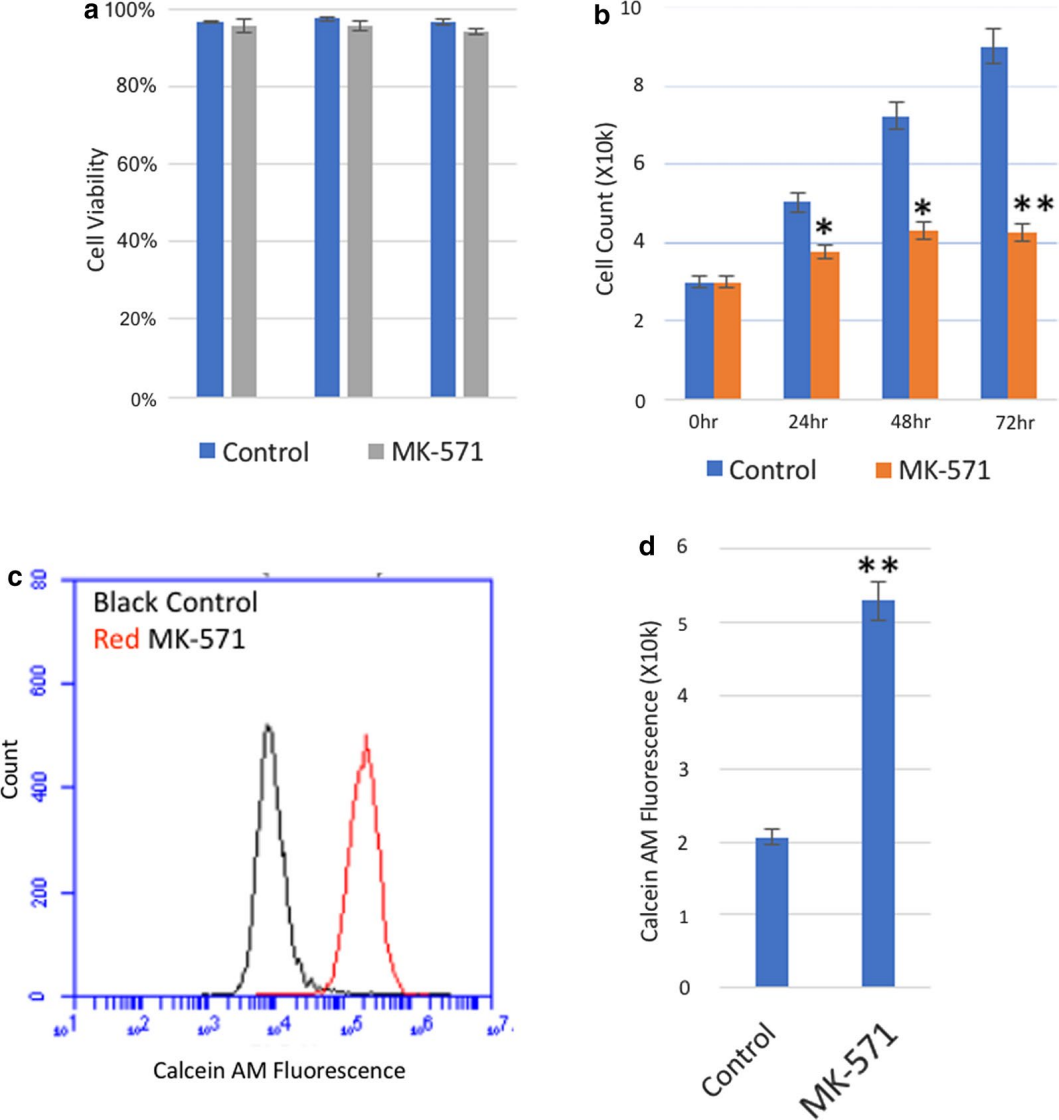
Functional inhibition of MRP1 transmembrane transport and GBM cell proliferation. T98G was treated with 25 µM of MK-571 for 72 h and untreated cells were used as a control. **a** The cell viability was measured by the Annexin-V assay and **b** cells were counted using the Trypan Blue assay. **c**, **d** At the 24 h time point, cells were incubated in 0.20 µM of Calcein AM staining solution at 37 °C. After incubation for 30 min, cell samples were immediately washed and analysed by flow cytometry to determine cellular uptake of Calcein AM. (*p < *0.0329*, **p < *0.0023 compared to untreated*) (*n *=* 4*; *mean ± standard deviation*)

To further elucidate the role of MRP1 in GBM cell proliferation, we exposed them to the functional inhibitor MK-571 which attenuates transmembrane transport of MRP-specific ligands. Unlike MRP1 siRNA, MK-571 did not inhibit the translation of MRP1 proteins. It was observed that exposure of cells to 25 μM MK-571 was sufficient to suppress cell growth without causing cell death (Fig. 3a, b). The inhibition of MRP1 transmembrane transport by 25 μM MK-571 was assessed by monitoring the export of intracellular Calcein AM, a fluorescent dye that is a substrate of MRP1, from GBM cells [40]. When MRP1 is functionally active in the cell, Calcein AM is exported by MRP1 as an indicator of its transporter function. We observed that the cells treated with MK-571 exhibited greater fluorescence signals from Calcein AM than untreated cells, indicating accumulation of Calcein-AM as a result of MRP1 inhibition (Fig. 3c, d).

### Decrease in proliferation rate and cell cycle arrest

**Fig. 4:**
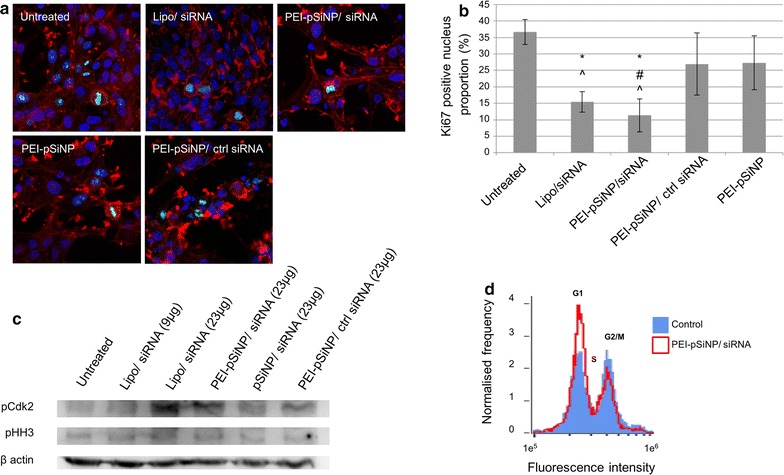
Effect of MRP1 silencing on the proliferative state of U87 cells. **a** Immunofluorescence of Ki67 imaged with confocal microscopy. **b** Quantitation of Ki67 positive cell proportion. (*^, #, ^^*p *< *0.05 as compared to untreated, PEI*-*pSiNPs loaded with ctrl siRNA, and PEI*-*pSiNPs, respectively*) (*n *= *3, mean ± standard deviation*). **c** Distribution of cells in G1/S and M phases as indicated by relative expression of phospho-tyr15 Cdk2 and phospho-ser10 histone H3 revealed by immunoblotting. **d** Distribution of cell cycle populations as illustrated by DNA content histogram

We further studied the proliferation of U87 cells under MRP1 silencing by monitoring the expression of Ki67, which plays an important role during mitosis, and hence serves as a marker for cell proliferation [41]. For all treatment groups, Ki67 expression in the nucleolus and chromosomes was observed during interphase and mitotic phase, respectively (Fig. 4a). However, the ratio between the Ki67 positive nuclei and the total population of nuclei was significantly reduced in Lipo/siRNA and PEI-pSiNP/siRNA treated U87 cells as compared to untreated controls, but not in PEI-pSiNP/ctrl siRNA (p = 0.275) and PEI-pSiNP treated cells (p = 0.127) (Fig. 4b). This is consistent with the observed reduction of EdU labelling and confirms that the reduced proliferation rate of U87 cells can be attributed to MRP1 knockdown.

This observation prompted us to study the effect of MRP1 silencing on cell cycle progression of U87 cells by probing the activation of cell cycle regulatory kinases. The elevated abundance of phosphorylated cyclin-dependent kinases 2 (pCdk2) and histone H3 (pHH3) in U87 cells implicate cells arresting at G1/S, and G2/M, respectively [42, 43]. By means of Western blot, we observed that the abundance of pCdk2 was higher in Lipo/siRNA and PEI-pSiNPs/siRNA treated U87 cells than for untreated cells by a factor of 2 and 1.8, respectively. The abundance of pHH3 was similar among the groups (Fig. 4c). Treatment with pSiNPs/siRNA and PEI-pSiNPs/ctrl siRNA resulted in only a slight pCdk2 elevation as compared to untreated cells by a factor of 1.2. Since the inhibitory phosphorylation on Cdk2 is maximal during G1/S [42], the elevated abundance of pCdk2 may suggest that the observed halt in proliferation is related to cells arresting at G1/S. A histogram of DNA content generated from flow cytometry also illustrates an increase in G1 cell population (from 34 to 56%), and a decrease of G2/M population (from 33 to 15%) upon MRP1 knockdown via PEI-capped pSiNP siRNA delivery (Fig. 4d). Collectively, the observed cell cycle arrest aligns with the results of cell cycle checkpoint proteins expression, EdU and Ki67 studies, which indicate an obvious reduction in cell proliferation upon MRP1 downregulation.

### MRP1 knockdown and proliferative state in tumours

**Fig. 5:**
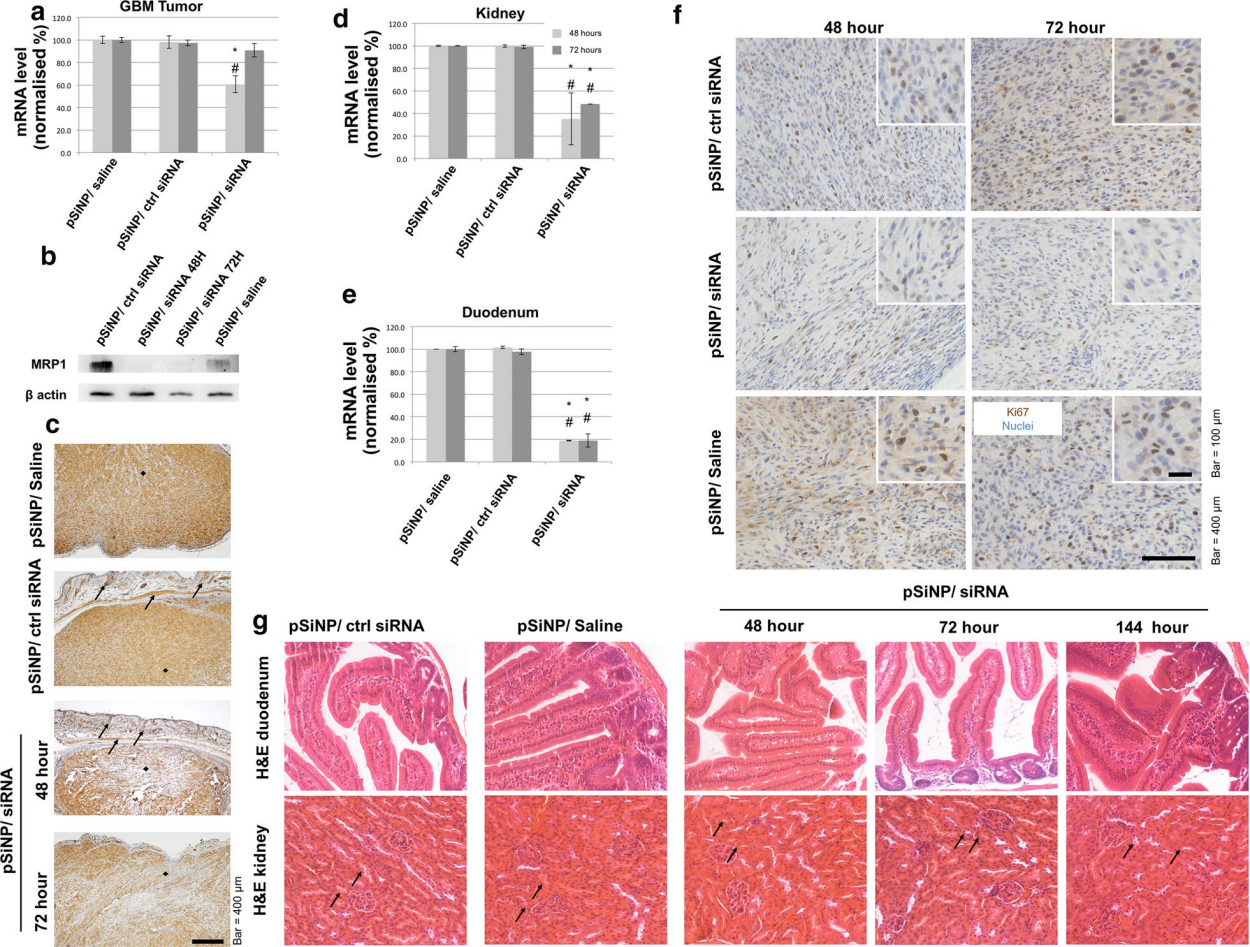
Effect of MRP1 silencing in vivo. MRP1 mRNA and protein expression level in tumours of mice being treated with siRNA-loaded PEI-pSiNPs at 48 and 72 h post-injection (intravenous) as revealed by **a** qRTPCR (*n *= *4*), **b** immunoblotting, and **c** immunohistochemistry. MRP1 mRNA expression level in **d** kidney (*n *= *2*) and **e** duodenum (*n *= *2*). **f** Immunohistochemistry of Ki67 expression in the tumour to reveal the proliferative state of U87 cells in mice receiving different treatments. **g** H&E staining of kidney and duodenum of mice treated with PEI-pSiNPs with MRP1 siRNA or ctrl siRNA, or none, revealing the histopathology of MRP1 downregulation in distal organs over 144 h (6 d) post-injection. (*^, #^*p *< 0*.05 as compared to controls pSiNPs/saline and pSiNPs/ctrl siRNA, respectively*)

The relationship between GBM proliferation and MRP1 downregulation was further investigated in a tumour bearing mouse model. CD-1 nude mice were subcutaneously (S.C.) inoculated with U87 cells, and the tumour growth kinetics were characterised (data not shown). Once the tumours reached a minimum size of 250 mm^3^, mice were treated with PEI-pSiNPs carrying either siRNA, ctrl siRNA, or saline intravenously for two consecutive days (2 mice and 4 tumours per group). mRNA extracted from tumours dissected at selected time points was analysed quantitatively for MRP1 expression. According to qRTPCR data, a reduction of MRP1 mRNA was observed at 48 and 72 h post-treatment, with the greatest reduction being 40% at 48 h (Fig. 5a). The expression began to recover between 48 and 72 h, reaching approximately 90% at 72 h. Reduction of MRP1 was also observed by immunoblotting at the protein level, consistent with gene expression level. The protein expression of MRP1 in tumours observed at 48 and 72 h post-treatment with PEI-pSiNPs/siRNA was reduced, by 82 and 65%, respectively, compared to the levels observed in tumours treated with PEI-pSiNPs delivering control siRNA (Fig. 5b). This demonstrates that the PEI-pSiNP successfully delivered siRNA to the tumour and yielded significant MRP1 knockdown.

To further evaluate the siRNA delivery and knockdown in vivo, we histologically compared the MRP1 protein distribution in tumours receiving PEI-pSiNPs/siRNA and control treatments. Immunohistochemical images correspond with qRTPCR and immunoblotting results, demonstrating a general downregulation of MRP1 in PEI-pSiNP/siRNA treated mice as compared to controls (Fig. 5c). Downregulation of MRP1 was also observed in mouse epidermis and dermis above the S.C. tumour in mice receiving MRP1 siRNA but not in control animals (Fig. 5c, arrows). Within the tumours, there was variation in downregulation of MRP1. Less MRP1 expression was seen in the GBM cells adjacent to the dermis, where blood vessels are abundant, while downregulation was less effective on the side adjacent to the peritoneum (Fig. 5c, squares). This gradient may reflect the penetration profile of PEI-pSiNPs into solid tumours.

The use of an untargeted delivery by nanoparticles allows investigation of the collateral downregulation of MRP1 in distal organs. We harvested kidney and duodenum, which also express MRP1 at physiological levels [44, 45]. qRT-PCR result suggested that the reduction of MRP1 mRNA in the kidney reached as much as 60% at 48 h post MRP1 siRNA treatment, and 55% (*n *= *2*) at 72 h (Fig. 5d). The reduction in the duodenum was even more pronounced, being 80% (*n *= *2*) at 48 h, with no recovery observed after 72 h post-treatment (Fig. 5e). Therefore, we conclude that MRP1 siRNA delivered in non-targeted nanoparticles, PEI-pSiNPs in this case, induces MRP1 knockdown in kidney and duodenum.

Ki67 expression in GBM tumours was also examined to study the proliferation potential of GBM cells experiencing MRP1 knockdown. Consistent with the in vitro results, Ki67 expression in the tumours of PEI-pSiNP/siRNA treated mice was reduced compared to controls (Fig. 5f). Such reduction in Ki67 was observed at both 48 and 72 h post-treatment, indicating that the proliferation retardation was sustained after nascent MRP1 mRNA, and protein levels started to restore. A longer time point analysis will be performed in future studies.

### Histopathology of MRP1 expressing organs

Since there is no definite conclusion to date as to the function of MRP1 in organs physiologically expressing MRP1, we performed histopathology analysis on the kidney and duodenum, in which MRP1 was shown to be collaterally downregulated, for signs of acute necrosis. The histological sections representing 48 h up to 144 h post-treatment were H&E stained. Histological analysis of kidney was focused on signs of acute tubular necrosis, which have been reported for nanotoxicity [46]. There were no observable differences between kidneys receiving ctrl-siRNA and MRP1-siRNA. All of them show no damage to endothelial cells, nor was an eosinophilic staining pattern or pyknosis of nuclei observed around proximal and distal convoluted tubule (Fig. 5g, arrows), indicating the absence of damage to tubular cells [47]. In the duodenum, we did not observe accumulation of lymphocytes in villi of both the controls and MRP1 silenced groups at all the time points. Neither did we observe differences in the population of goblet cells. These results indicate that there was no sign of acute duodenitis [48].

## Discussion

MRP1 expression has been identified in a variety of tumours and is being comprehensively studied as a therapeutic target for chemosensitisation. Various modulators have been pursued over the last two decades [11, 12]. Combination therapy has already shown effectiveness in vitro and in vivo in lung carcinoma [10] and breast cancers [49]. Clinical trials exploring MRP1 knockdown with a small molecule modulator in patients with breast, lung, bowel, melanoma, renal and ovarian cancers are ongoing [50].

GBM remains poorly treated, therefore exploring the promise and effect of MRP1 downregulation using a nanoparticle delivery approach has merit. To date, there are only a handful of reports based on in vitro trials. For example, patient derived GBM cells were chemosensitised in vitro using an MRP1 inhibitor [13]. A study by Tivnan et al. tested chemosensitisation with GBM cells derived from relapsed patients, demonstrating some effectiveness in this setting [8]. Additionally, various nanoparticle delivery systems, including ours, have been developed to deliver modulators of MRP1 in glioma cells [51, 52]. However, in vivo effectiveness and effects on other cells remains to be investigated. Although the correlation between MRP1 expression and more aggressive phenotype of cancer has been observed [53], phenotypic effects of the knockdown of MRP1 remain unknown.

In this study, we used PEI-capped pSiNPs as a siRNA delivery vehicle in vitro and in vivo in order to study the knockdown and phenotypic changes, both in tumour and collateral organs. The reason for choosing the pSiNP delivery vehicle for MRP1 siRNA is due to a number of inherent desirable properties including high loading capacity, biodegradability and biocompatibility. Furthermore, we have previously documented the successful in vitro knockdown of MRP1 with siRNA delivered using pSiNPs [16, 23]. The PEI-pSiNPs in this study were of an average size of 170 nm, smaller than the average size of tumour vasculature ranging from 380 to 780 nm [54]. Additionally, nanoparticles at 100 nm range have generally proven to be long-lasting in the circulation [34]. We optimised the release profile for in vivo delivery through optimising the pSiNP capping and washing procedure. The percentage siRNA released after 24 h was found to be only 10%, while 80% of the siRNA was released between 24 and 48 h. In view of previous studies that have shown PEI nanoparticles, such as JetPEI, to facilitate distribution to the respective organs at 24 h [39], we believe that the release profile of the PEI-pSiNPs used here would prevent immature burst release, and thus deliver most of the siRNA into the tissues. As compared to other reported nanoparticles of comparable size, chitosan nanoparticles showed siRNA burst release before 24 h with only 10% siRNA being released between 24 and 48 h [55], whilst for PLGA nanoparticles release of 50% of the loaded amount was reported before 24 h, and only 10% was released between 24 and 48 h [56].

The superior siRNA retention for the first 24 h is thought to be due to the electrostatic interaction between PEI and siRNA and the packing of the siRNA into the pores of the pSiNPs [57]. The PEI capping also facilitated an obvious improvement of cell uptake in GBM cells as expected. Whilst the risk of cytotoxicity associated with branched PEI gene delivery due to the charge density has been a concern for clinical translation [58], cytotoxicity was not observed with our pSiNP delivery. This is consistent with our previous study in terms of a lack of observable apoptotic and cytotoxic phenotypes [32]. Although the mechanism of such diminished toxicity remains unclear, a similar observation was reported by Yuen Shan et al., who evaluated PMMA nanoparticles coated with branched 25 kDa PEI for gene delivery and showed efficient transfection and low cytotoxicity [59].

MRP1 protein expression was downregulated with MRP1 siRNA but not with the scrambled siRNA sequence, indicating specific MRP1 effects. While the knockdown efficiency of siRNA delivered with lipofectamine (51%± 4%) was observed to be higher than that of PEI-pSiNPs (30 ± 9%) in vitro, 30% of downregulation was found to be sufficient to sensitise U87 cells to doxorubicin and reduce the viability by 70% as compared to exposure to doxorubicin only. While this level of downregulation is comparable to our previous in vitro study [16], the delivery vehicle used here did not cause cytotoxicity by itself, indicating that it is potentially applicable for systemic delivery.

The consistent decrease in total cell counts in MRP1 siRNA transfected U87 cells, without an increase in apoptotic cell death was further investigated. We demonstrated that the proliferation of GBM cells was proportional to MRP1 expression level by means of an EdU incorporation assay and Ki67 immunofluorescence, indicating that U87 cells have a much slower proliferation rate when MRP1 is silenced. Similar studies of MRP1 downregulation in neuroblastoma cells using antisense expression vectors revealed spontaneous cell death and a reduction in cell proliferation [60, 61]. For ovarian carcinoma cells, Mahdizadeh et al. characterised the effect of saffron extract-crocin exposure and observed both MRP1 silencing, cytotoxicity, and a reduction in cell proliferation [62]. However, the mechanism of the observed proliferation retardation in those cancer types was not further investigated. In contrast, we observed no glioma cell death upon transfection.

Here, the cell cycle arrest at G1/S observed in GBM was demonstrated to be associated with MRP1 silencing by determining the relative abundance of phosphorylated Cdk2 and the increase in G1 cell population. This observation differs from multidrug transporter P-glycoprotein (Pgp) knockdown, which was shown to cause cell cycle arrest at G2/M in leukaemia cells with apoptosis induction [63]. Since MRP1 and Pgp transport different substrates across the plasma membrane [64], we speculate that the effect of MRP1 knockdown on proliferation is mediated through the inhibition of transmembrane trafficking. The functional inhibition of MRP1 using MK-571 also resulting in attenuation of proliferation appears to support such speculation.

Since PEI-pSiNP delivery of siRNA is a biocompatible and versatile platform, it allowed us to characterise the MRP1 knockdown approach and to validate the decrease in proliferation of GBM in vivo. We demonstrated that the siRNA delivery resulted in MRP1 downregulation at both mRNA and protein level. The observed siRNA-induced knockdown indicates that the siRNA was delivered into the cytoplasm where it forms RNA-induced silencing complex (RISC) with the complementary mRNA before the translation [65]. Hence, it can be concluded that the PEI-pSiNPs successfully accumulated and delivered the siRNA into the subcutaneous xenograft GBM tumour.

The MRP1 downregulation was observed throughout most of the tumour under histological study, indicating that the size of PEI-pSiNPs (~ 170 nm) was generally able to penetrate the GBM tumour mass and deliver the payload. The penetration of nanoparticles into solid tumour depends on the size and surface chemistry of the nanoparticles, and also the architecture of the tumour [66]. Although S.C. tumours do not replicate the complexity of orthotropic GBM, such as the requirement to transverse the BBB, our results serve as the first step translating the promise of the MRP1 silencing approach reported based on in vitro studies under GBM-specific in vivo tumour microenvironment. For example, this approach could be promising for coating the resected bed of the tumour at the completion of surgery and to use an Omaya reservoir for MRP1-siRNA delivery to minimise GBM recurrence through its anti-proliferative and chemosensitising properties, since the BBB is not intact at glioblastoma progression, and at post-surgery [67–69].

For the effectiveness of the delivery to early stage of glioma, where the BBB is intact, such approach remains to be further optimised and evaluated in suitable orthotropic models.

It has been reported that the expression of Ki67 in brain cancers correlates with histological malignancy grade in all glioma subtypes [70]. It is also clinically accepted that Ki67 generally reflects the cancer aggressiveness, as its function is closely linked to cell division [41]. The diminished Ki67 positive proportion in tumours of the mice receiving PEI-pSiNPs/siRNA indicates that MRP1 downregulation correlates with GBM proliferation, in agreement with the in vitro result. Thus, we believe our observations highlight the potential that suppression of GBM proliferation associated with MRP1 silencing could be exploited to suppress the progression of the residual GBM, in parallel to chemosensitising the tumour.

Biodistribution of the cationic nanoparticle is well-documented to indicate accumulation in the liver, spleen, kidney, and lung [71], and both kidney and duodenum tissues are known to express MRP1 as their physiological phenotype [44, 45]. We observed significant downregulation of MRP1 in these two organs, which was even more long-lasting than the silencing in the S.C. GBM tumour, which may be due to the expected accumulation profile of the non-targeted PEI-pSiNPs. It is reported that the physiological function of MRP1 in kidney and digestive system is related to protection from natural toxins, cholehepatic and enterohepatic circulation of bile, and in the protection of the biliary tree tissues against toxic bile constituents [72]. However, no histopathological signs that would indicate tissue damage such as necrosis in these two organs was observed over 6 days post-treatment. Since MRP1 knockdown in tumour and proliferation inhibition were observed as early as 48 h post-injection and sustained despite nascent mRNA started recovering, this may indicate a possible treatment window. Future studies should investigate the collateral damage of MRP1 siRNA plus cytotoxic drug co-treatments in other organs, such as heart which is susceptible to oxidative damage, while MRP1 knockdown would further decrease the tolerance in normal organs to cytotoxic drugs [73]. In addition, targeted delivery of MRP1 siRNA should be explored in follow-up studies to minimise the undesired silencing of MRP1 in other organs. Given that MRP1 expression also causes radiation resistance, we speculate that localised delivery at the site of primary tumour resection may provide a means of sensitisation towards chemo- and radiotherapy and obviate the need for systemic exposure and potential toxicity from MRP1 downregulation in other organs.

This study highlights the relationship between MRP1 and GBM cell proliferation, and provides insights into the relationship between MRP1 expression and malignancy grade. In addition, we demonstrated that MRP1 silencing by employing PEI-pSiNP delivery of siRNA alone would reduce the proliferation rate of GBM cells by attenuating the cell cycle at G1/S, without cytotoxic drug co-treatment, indicating the importance of MRP1 expression in the biology of GBM. Since the effect of siRNA subsides over time mainly due to the dilution of intracellular siRNA owing to cell division [74], the proliferation attenuation effect associated with MRP1 silencing may intensify the therapeutic effect of this approach. MRP1 knockdown of GBM was demonstrated here for the first time in vivo, and the proliferation attenuation was also observed in the tumour, highlighting the promise of MRP1 as a treatment to GBM.

## Conclusion

Chemoresistance in GBM, partially mediated by MRP1 overexpression in the brain, renders GBM a fatal disease that is notoriously difficult to eradicate. Gene therapy aimed at MRP1 silencing has been showing promising results in sensitising other drug-resistant cancers while its effect on brain cancers have not been thoroughly investigated. Here, we demonstrated that the MRP1 silencing using pSiNP-based delivery of MRP1 siRNA not only drug-sensitised, but also inhibited GBM cell proliferation by arresting cell cycle at G1/S on its own. The reduction in proliferation rate was independent of the siRNA delivery method. This effect may be mediated via transmembrane transporters as suggested by a MRP1 functional inhibition assay. Through the use of PEI-capped pSiNP delivery of siRNA, we established MRP1 silencing in GBM tumours in mice. Consistently, the reduction of ki67 positive staining in GBM correlated with MRP1 silencing in the tumour. MRP1 siRNA delivery was not targeted to tumours and hence also showed silencing in MRP1-expressing organs such as kidney and duodenum. However, no histopathological signs were observed. In conclusion, this study illustrated, for the first time, the correlations between MRP1 expression and GBM proliferation. This provides insights into residual GBM eradication through highlighting the potential that, apart from chemotherapeutics sensitising, MRP1 silencing itself may deliver therapeutic effects by attenuating the growth of GBM.

## Additional files

**Additional file 1: Figure S1.** FTIR-ATR surface chemical analysis of pSiNP indicating the completion of thermal hydrocarbonisation of pSiNP.

**Additional file 2: Figure S2.** Chemosensitisation of which GBM cells as indicated by the viability of cells treated by pSiNP/siRNA, or DOX, or combination treatment, or untreated.
