# Supplementary figures and images for: Delivery of siRNA in vitro and in vivo using PEI-capped porous silicon nanoparticles to silence MRP1 and inhibit proliferation in glioblastoma
Source: J Nanobiotechnology. 2018 Apr 13;16:38. doi: 10.1186/s12951-018-0365-y (PMC5898074; doi:10.1186/s12951-018-0365-y)

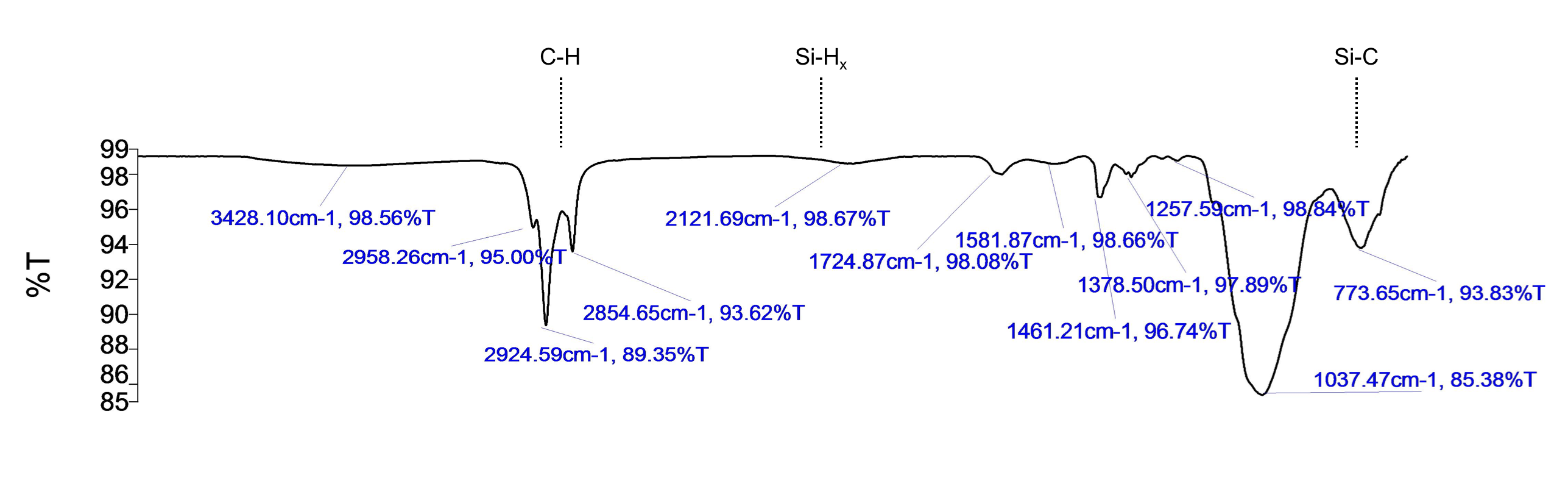

Supplement: Supplementary file 1 — Additional file 1: Figure S1. FTIR-ATR surface chemical analysis of pSiNP indicating the completion of thermal hydrocarbonisation of pSiNP. [file 12951_2018_365_MOESM1_ESM.png]

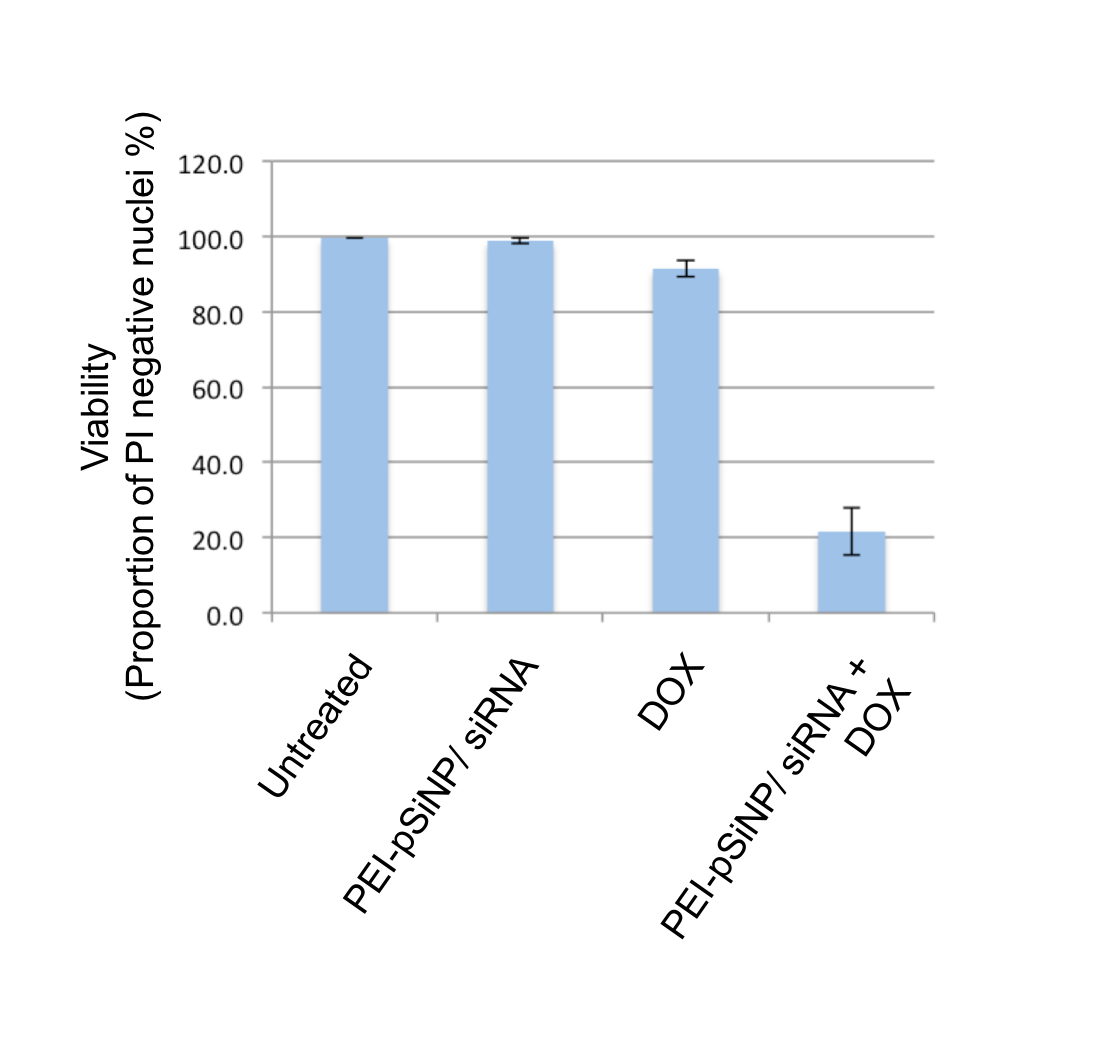

Supplement: Supplementary file 2 — Additional file 2: Figure S2. Chemosensitisation of which GBM cells as indicated by the viability of cells treated by pSiNP/siRNA, or DOX, or combination treatment, or untreated. [file 12951_2018_365_MOESM2_ESM.png]
